# Polyacylurethanes as Novel Degradable Cell Carrier Materials for Tissue Engineering

**DOI:** 10.3390/ma4101705

**Published:** 2011-10-06

**Authors:** Danijela Jovanovic, Frans V. Roukes, Andrea Löber, Gerwin E. Engels, Willem van Oeveren, Xavier J. Gallego van Seijen, Marja J.A. van Luyn, Martin C. Harmsen, Arend Jan Schouten

**Affiliations:** 1 Department of Polymer Science, Faculty of Mathematics and Natural Sciences, Zernike Institute for Advanced Materials, University of Groningen, Nijenborgh 4, 9747 AG Groningen, The Netherlands; E-Mails: d.jovanovic@rug.nl (D.J.); frans_roukes@hotmail.com (F.V.R.); 2 Institute for Bioengineering, Martin-Luther University, Halle 06108, Germany; E-Mail: andrea_loeber@gmx.de; 3 Department of Biomedical Engineering, University Medical Centre Groningen, University of Groningen, A. Deusinglaan 1, building 3215, FB40, 9713AV Groningen, The Netherlands; E-Mails: g.e.engels@med.umcg.nl (G.E.E.); w.van.Oeveren@med.umcg.nl (W.O.); 4 Department of Pathology and Medical Biology, University Medical Centre Groningen, University of Groningen, Hanzeplein 1, 9713 GZ, Groningen, The Netherlands; E-Mails: x_gallego@hotmail.com (X.J.G.S.); m.j.a.van.Luyn@med.umcg.nl (M.J.A.L.); m.c.harmsen@med.umcg.nl (M.C.H.)

**Keywords:** biodegradable polymers, tissue engineering, biomedical polyurethanes, hydrolytic degradation, acylurethanes, blood compatibility, endothelial cells

## Abstract

Polycaprolactone (PCL) polyester and segmented aliphatic polyester urethanes based on PCL soft segment have been thoroughly investigated as biodegradable scaffolds for tissue engineering. Although proven beneficial as long term implants, these materials degrade very slowly and are therefore not suitable in applications in which scaffold support is needed for a shorter time. A recently developed class of polyacylurethanes (PAUs) is expected to fulfill such requirements. Our aim was to assess *in vitro* the degradation of PAUs and evaluate their suitability as temporary scaffold materials to support soft tissue repair. With both a mass loss of 2.5–3.0% and a decrease in molar mass of approx. 35% over a period of 80 days, PAUs were shown to degrade via both bulk and surface erosion mechanisms. Fourier Transform Infra Red (FTIR) spectroscopy was successfully applied to study the extent of PAUs microphase separation during *in vitro* degradation. The microphase separated morphology of PAU1000 (molar mass of the oligocaprolactone soft segment = 1000 g/mol) provided this polymer with mechano-physical characteristics that would render it a suitable material for constructs and devices. PAU1000 exhibited excellent haemocompatibility *in vitro*. In addition, PAU1000 supported both adhesion and proliferation of vascular endothelial cells and this could be further enhanced by pre-coating of PAU1000 with fibronectin (Fn). The contact angle of PAU1000 decreased both with *in vitro* degradation and by incubation in biological fluids. In endothelial cell culture medium the contact angle reached 60°, which is optimal for cell adhesion. Taken together, these results support the application of PAU1000 in the field of soft tissue repair as a temporary degradable scaffold.

## 1. Introduction

Degradable polymers are preferred candidates for designing therapeutic devices to treat missing or damaged soft tissues. Being FDA approved, polycaprolactone (PCL) has been intensively investigated as temporary scaffold biomaterial. However, PCL suffers from significant drawbacks. PCL is found to degrade very slowly both *in vitro* and *in vivo*, with almost no mass loss or decrease in molar mass for at least 6 months of degradation [1,2]. In addition, in order to achieve good mechanical properties, the molar mass of PCL has to be relatively high which leads to an increase in crystalline fraction of this semi-crystalline polyester. The latter might cause an obstacle for healthy regeneration *in vivo* [3,4].

Our aim was to develop a biomaterial with tunable (degradation) properties and surface characteristics that allow for cell adhesion to serve as temporary support in soft tissue regeneration.

Polyurethanes possess good mechanical properties and blood compatibility, which have made them attractive for their use for manufacture of biomedical devices [5]. The possibility to alter their mechanical properties by changing the ratio between the constituent components, so-called soft segments (polyether, polyester, and polycarbonate) and hard segments (aliphatic or aromatic diisocyanates), renders them very useful in a variety of materials with different requirements.

To the best of our knowledge *Endo et al.* [6] have described polyacylurethanes in literature for the first time in 1994. The polymers were prepared by the polyaddition of bis(N-acyl isocyanates) with low molecular weight diols and polyether diols. The most interesting feature of this polymerization was the high reactivity under mild conditions of the bis(N-acyl isocyanates) towards the polyether diols without the use of additional catalysts.

In a patent of 1995 *Yabuta* and *Urano* claimed the preparation of polyacylurethanes where the diol could be a polycaprolactone prepolymer, introducing this class of polymers in the area of thermoplastic elastomers [7]. However, the procedure described involved quite high temperatures and long reaction times and resulted “preferably” in rather low molar mass polymers. Apparently also preferred was the use of self-condensing unsymmetrical monomers containing only one acylisocyanate group. The polymers have been characterized as easy-degradable.

Recently, polyacylurethanes (PAUs) have been developed in our laboratory [8,9]. These materials were produced without the use of any toxic catalysts of which remnants might limit biomedical use. Polyacylurethanes are synthesized with terephthaloyl diisocyanate (TPHDI), which is expected not only to enhance materials mechanical properties due to improved micro-phase separation [10], but is also expected to result in non-toxic degradation products [11]. The acyl functionality contributes to the greater reactivity of TPHDI [12] and is expected to hydrolyze relatively fast [13].

The goal of this research was to assess the hydrolytical degradation of PAUs, with an accent on the mechanism of PAU hydrolysis and microphase separation, and to evaluate these materials for application in regenerative medicine as a biodegradable scaffold. Describing the degradation of PAUs *in vitro* will be used as a starting point to explain its *in vivo* behavior in the future.

In this study, we show the results of the degradation study of PAUs with different lengths of the oligo(*ε*-caprolactone) soft segments (Number average molar mass = 1000, 1500 and 2000 g/mol, PDI = 1.69, 1.82 and 1.90, respectively). PAU characterization included monitoring mass loss, molar masses, and thermal and surface properties of PAUs upon a degradation period of 80 days. The microphase separation of PAUs was analyzed by utilizing Fourier Transform Infra Red (FTIR) spectroscopy. PAU1000 (molar mass of the oligocaprolactone soft segment = 1000 g/mol) was selected as the potential scaffold material to be used in regenerative medicine due to its optimal behavior during hydrolytic degradation and suitable micro-phase separation behavior. On this particular PAU composition, a series of biological assays were performed to test cytotoxicity and haemocompatibility. Human umbilical vein derived endothelial cells (HUVEC) were used for *in vitro* cell adhesion and proliferation experiments. Since both cell adhesion and blood interactions with biomaterials take place in the material-protein (cell) interface [14,15], surface properties of PAU1000 were also investigated.

## 2. Results and Discussion

### 2.1. Mass Loss and Molar Mass Change

Polyester urethanes are believed to undergo hydrolytic degradation via two different mechanisms identified as bulk degradation and surface erosion [16,17]. Bulk degradation is characterized by an overall decrease of molar mass if random chain scission is occurring. Surface erosion is the process where hydrolysis removes only polymeric chains from the outer layer of the material and this leaves the bulk of the material untouched [16]. Surface erosion is favored for many applications of polymeric biomaterials (e.g., controlled drug delivery), because the material properties remain virtually intact since degradation proceeds through removal of very thin layers of the material.

**Figure 1:**
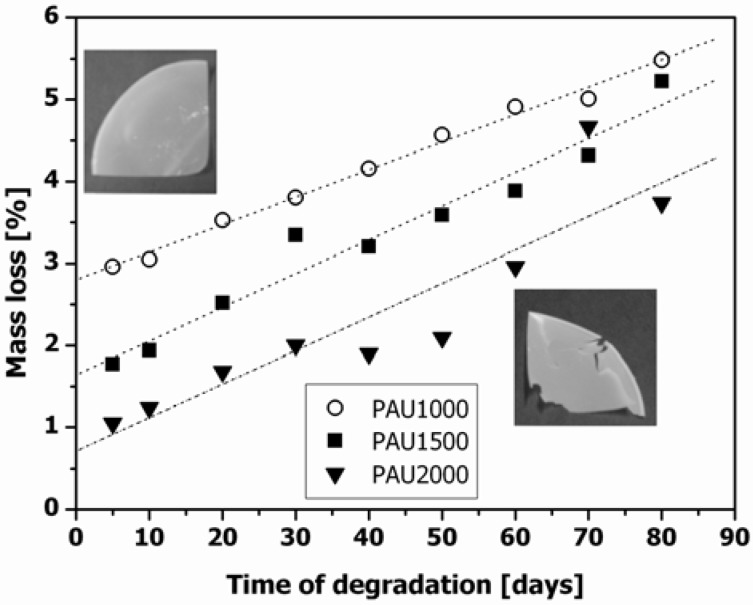
Mass loss of polyacylurethane (PAU) films with different soft segment lengths at 37 °C in phosphate buffer (pH = 7.2). (○) PAU1000; (■) PAU1500 and (▼) PAU2000.

For all three PAUs, mass loss showed a similar pattern, with a 2.5–3.0 wt% decrease in mass at the final time point (Figure 1), which is relatively high, especially when compared to other PCL-based materials that are intended for similar applications and which show almost no mass loss even for more than a year [1,18]. The apparent high mass loss at the first incubation time point (5 days) can be attributed to different sample manipulation of the incubation samples compared to the non-incubated samples. During preparations for the degradation study (see Section 3.4) prior to weighing, samples were exposed to air and thus may have absorbed water. Therefore, by calculating the linear fit, we only compared the mass loss trends of the three PAUs and calculated the mass loss at the end of the examination period (day 80). Since the potential impurities and residual non-reacted material were removed by means of Soxhlet extraction with n-hexane, the observed mass loss upon incubation can only be ascribed to PAU degradation. The PAU films changed from flexible to brittle and fragile with the course of degradation (Figure 1).

**Figure 2:**
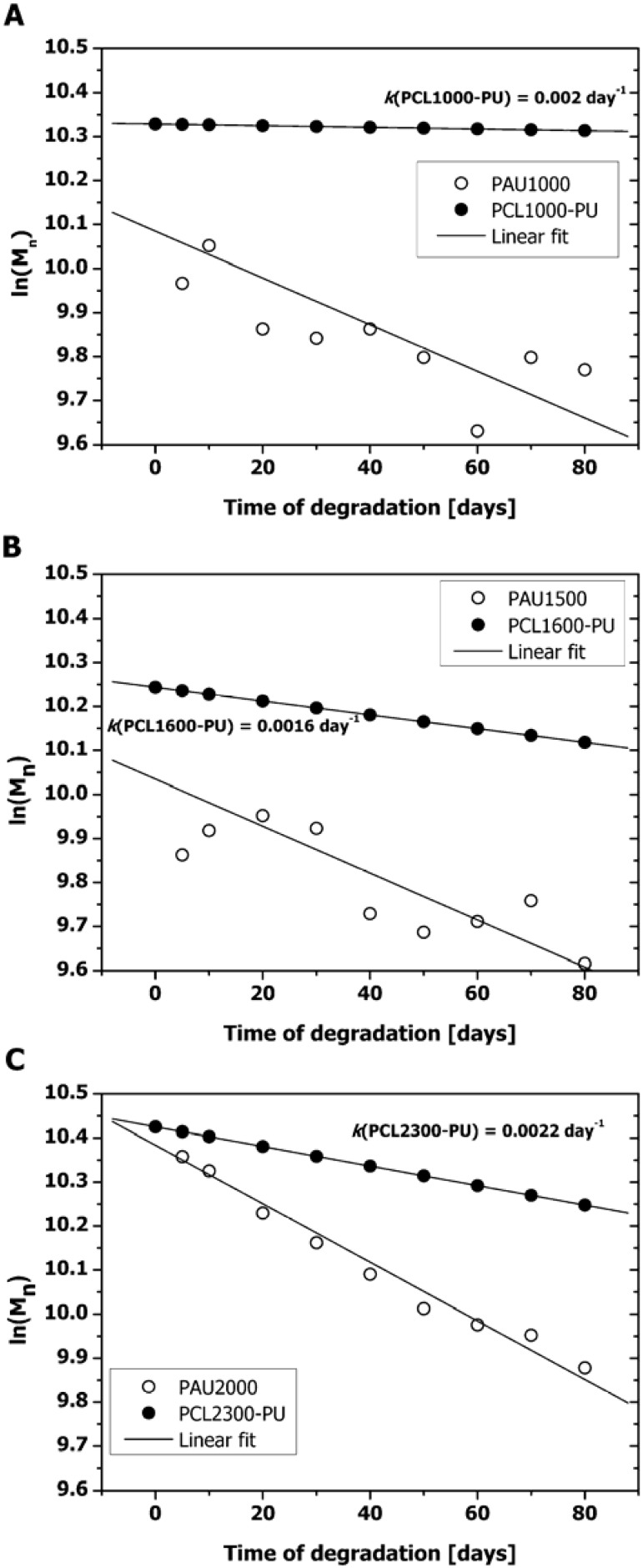
Decrease of PAUs’ molar mass in the course of hydrolytic degradation. (**a**) PAU1000 compared to PCL1000-PU; (**b**) PAU1500 compared to PCL1600-PU; (**c**) PAU2000 compared to PCL2300-PU. Data set for PCL-PUs was calculated assuming the same *M_n_^0^* and oligodiol molar mass of corresponding PAUs and hydrolysis rate constant (*k*) value for Polycaprolactone (PCL)-PUs as given by Heijkants *et al.* [18].

The degradation was accompanied by molar mass decrease, indicating that chain scission in bulk had occurred additionally. The molar mass of degraded PAUs decreased approximately 32% for PAU1000 and PAU1500, to 38% for PAU2000 compared to the non-degraded samples (data not shown). It is generally believed that *in vitro* degradation of polyester urethanes proceeds via random scission of the ester bond of the polyester soft segment. Pitt *et al.* [19] derived the following equation to describe the *M_n_* decrease during ester hydrolysis:

(1) $$\frac{1}{Mn}=\frac{1}{{Mn}^{0}}\times e^{k\times t}$$

or in its linearized form:

(2) $$ln(Mn)=ln(Mn^{0})-k\times t$$

where *M_n_* [gmol^−1^] is the number average molar mass of the polymer at any time point, *M_n_^0^* is the initial number average molar mass [g mol^−1^], *k* is the ester hydrolysis rate constant [day^−1^] and *t* is the degradation time [day]. If we assume that the above described model is applicable for PAU degradation, then values of ln*(M_n_^0^)* of PAUs as a function of *t* can be described by a linear function (Figure 2).

**Figure 3:**
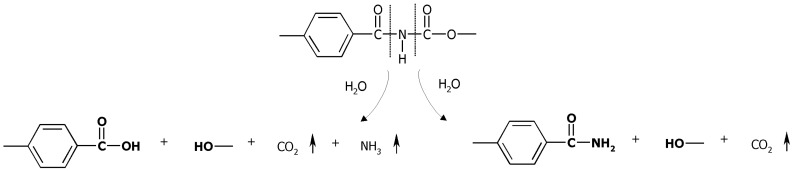
Hard segment hydrolysis.

However, when fit parameters were analyzed, *r^2^* value for PAU1000 and PAU1500 fit were ~0.6 which indicated a poor fit. This further implied that the PAU degradation proceeded not only via scission of ester bonds of the PCL soft segment. Water can attack the following functional groups: Ester groups of the soft segments, acyl groups of the hard segments and urethane groups of the hard segments (Figure 3). As a result, aliphatic carboxylic acids, aromatic carboxylic acids, aliphatic alcohols and primary amides can be formed. Both hydrolysis of the soft segment esters and acyl urethane groups are acid catalyzed and expected to contribute greatly to PAU degradation.

The observed deviation of the proposed pseudo-linear autocatalytic model and degradation of PAUs originates from the fact that the basic assumptions for this model were not fulfilled. The autocatalytic model was derived based on the following assumptions: (1) extent of chain scission is small, (2) only ester bonds hydrolyze and (3) there is no mass loss [19]. PAUs did loose mass upon degradation and in addition to the ester groups of the soft segment, acylurethane groups also might have hydrolyzed. Even though they were both composed of the same molar mass of the PCL soft segment, PAUs degraded much faster than PCL-PUs based on a BDI hard segment [18] (Figure 2). This can be explained by the chemical nature of the acylurethane moiety and the lower degree of microphase separation of PAUs compared to the PCL-PUs (see Section 2.3 for details).

The combination of mass loss and decrease of molar mass indicates that the hydrolytic degradation of PAUs occurs with combined bulk and surface erosion mechanisms.

### 2.2. Thermal Properties of PAUs Upon Degradation

**Figure 4:**
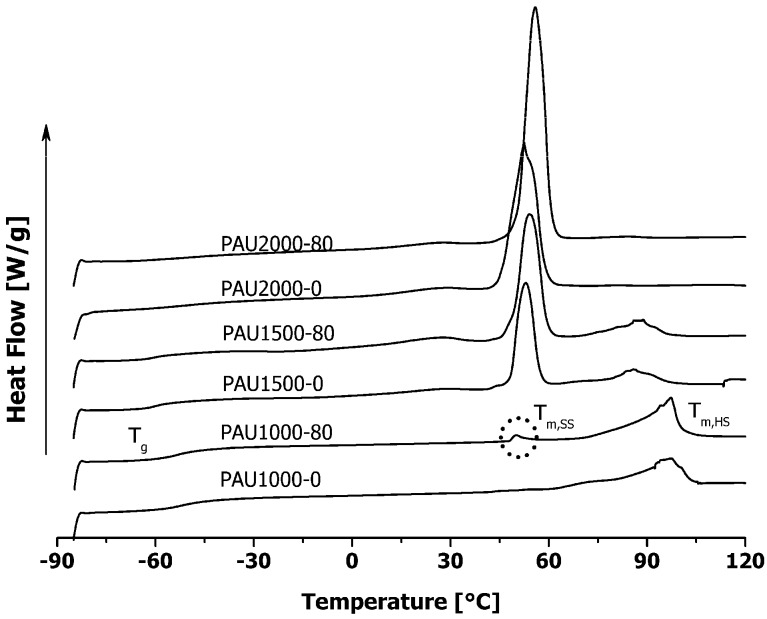
Thermal properties of PAUs upon degradation at 37 °C in phosphate buffer solution (pH = 7.2). Heating endotherms (1st heating scan) of PAUs with different PCL-oligodiol lengths non-degraded (0 days) and at the end of the degradation period (80 days). *T_g_*-glass transition temperature; *T_m,,SS_*-soft segment melting temperature; *T_m,HS_*-hard segment melting temperature.

Three PAUs with different PCL-oligodiol molar masses (PAU1000, PAU1500 and PAU2000) exhibited different melting endotherms (Figure 4).

Analysis of the PAUs before the *in vitro* degradation revealed a microphase separated structure in the case of PAU1000 and PAU1500, characterized by a hard segment melting peak and the glass transition temperature (*T_g_*) of the soft segment (Figure 4). PAU2000 was not microphase separated, showing only a melting peak originating from crystalline PCL-soft segment, indicating that the preferred mechanical properties typical for polyurethanes were virtually absent.

**Figure 5:**
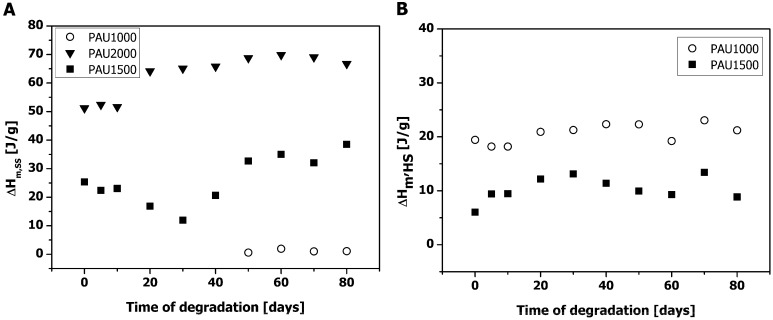
Soft segment and hard segment melt enthalpies of PAUs upon degradation. (**a**) Soft segment melt enthalpy (ΔH_m,SS_); (**b**) Hard segment melt enthalpy (ΔH_m,HS_).

Both PAU1500 and PAU2000 contained a relatively high fraction of crystalline PCL-soft segment (no *T_g_* detectable of the soft segment of PAU2000) which could retard the hydrolytic degradation both *in vitro* and *in vivo*. The appearance of the soft segment melting peak in PAU1000 from day 50 (Figure 4 & Figure 5) on is a result of the chain scission and increased mobility of PCL-soft segments. Similar results have been observed earlier by different research groups [1,2,20].

However, the amount of crystalline PCL even after 80 days of degradation was negligible (Figure 5a) and it was not expected to influence PAU1000 degradation to a great extent. The soft segment crystallinity of PAU2000 gradually increased with incubation, most probably because some chain scission had occurred in the amorphous phase of the PAU2000, allowing for higher chain mobility. In addition, the overall mobility of the chains might have been facilitated by the long incubation (annealing) in the humid environment at 37 °C.

PAU1000 exhibited the highest (bulk) hard segment crystallinity (Figure 5b), which remained constant during the course of degradation.

### 2.3. Microphase Separation of PAUs Prior to Degradation (Trans-FTIR)

Since hydrolytic degradation can only take place in the amorphous fraction of the polymer, the degree of microphase separation is one of the key factors that determine the hydrolysis rate of segmented polyester urethanes. To be able to determine which fraction of the hard segment was crystalline from the total urethane present (as a measure of the extent of the microphase separation), an enthalpy of melting of the 100% crystalline hard segment should be known. Since this was not the case, we attempted to calculate the extent of the microphase separation from the FTIR deconvolution analysis of the carbonyl absorption region of the PAUs. The carbonyl region was found to be suitable for this determination as described by *Pretsch et al.* [21]. Since only PAU1000 and PAU1500 exhibited microphase separation, PAU2000 was excluded from the FTIR analysis.

**Figure 6:**
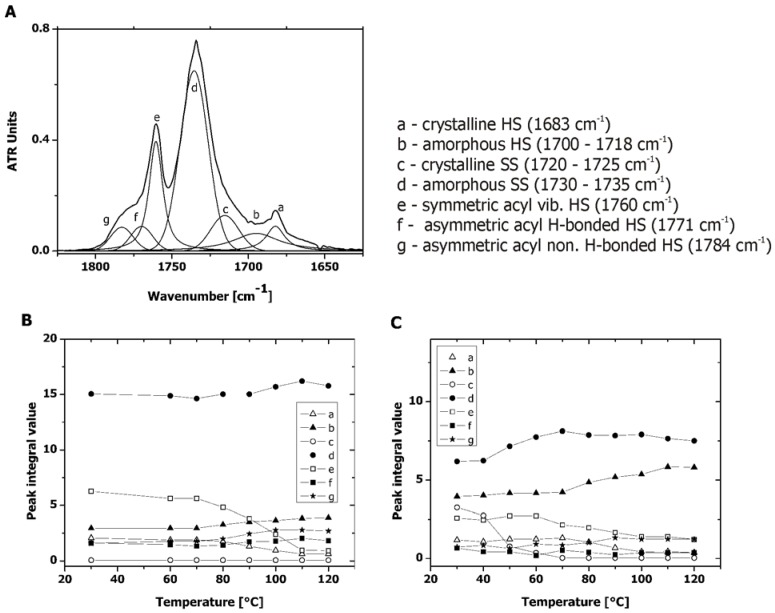
Carbonyl region of PAUs (transmission Fourier Transform Infra Red (FTIR)) prior to degradation. (**a**) Assignment of peaks of the PAU1000 carbonyl region curve fit; (**b**) PAU1000–integral values of peaks with temperature increase; (**c**) PAU15000–integral values of peaks with temperature increase.

Bearing in mind that H-bond associated C=O always appears at the lower wavenumbers [21,22], and correlating the deconvolution results with the DSC observations (Figure 5), overlapping peaks in the C=O region were assigned as denoted in Figure 6. As already known from the literature, upon heating, dissociation of H-bonding occurs, followed by the disorder of the hard segment (HS) and soft and hard segment mixing [23,24]. The disruption of the HS crystallinity led to the decrease in the crystalline HS peak in both PAU1000 and PAU2000 (Figure 6). The decrease of the HS crystallinity was accompanied by the increase of the amorphous HS portion (Figure 6). As already observed by DSC, PAU1000 did not contain PCL soft segment (SS) in the crystalline form. The SS crystallinity of PAU1500 decreased upon heating, which resulted in the majority of the SS to be amorphous (Figure 6).

**Scheme 1:**
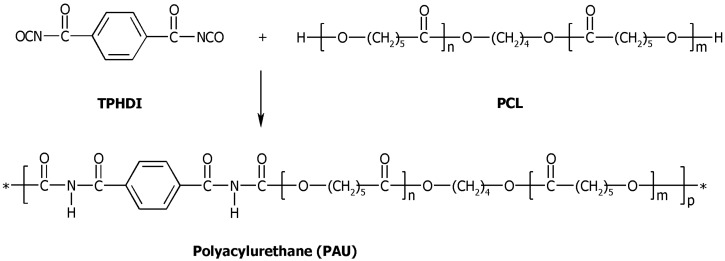
Synthesis of polyacylurethanes. Terephthaloyl diisocyanate (TPHDI) and poly(*ε*-caprolactone) (PCL) are reacted without a catalyst to yield polyacylurethane (PAU).

If we observe the molecular structure of the PAUs (Scheme 1), we could suspect that the acyl carbonyls and the phenyl ring could engage in a resonance interaction, which would further result in coplanar intermolecular orientation and four different conformations (two *cis* and two *trans*). Acyl carbonyls would than rotate relatively freely around the single bond and the differences in the levels of vibration energies specific for the different conformers would be small. However, if such a system gets fixed in any way, the difference in energy levels of the conformers would become larger, which would result in the splitting of the acyl carbonyl band. *Sun et al.* observed this phenomenon by analyzing chemically similar polymers, poly[di(butyl) vinylterephthalate] (PDBVT) [25]. Due to the attachment of the phenyl ring to a polyvinyl backbone, the energy levels became further apart, leading to acyl carbonyl band splitting [25].

The acyl carbonyl group of the PAUs is located next to the urethane bond, and participates in the hydrogen bonding (H-bonding) of the HS. Since H-bonding of the HS prefers certain conformations above other ones, rotation of the acyl carbonyls from both sides of the phenyl ring becomes restricted. Temperature increase results in the disturbance of the H-bonds of the HS in the crystalline phase, which is detected as a decrease of peaks representing acyl carbonyl in the crystalline form, and an increase of peaks originating from acyl carbonyl in the amorphous form. Since associated carbonyls always appear at lower frequencies than their non-associated counterpart [22], the peak at 1771 cm^−1^ has been assigned to H-bonded acyl carbonyl in the amorphous phase (Figure 6). The fact that these peaks increased with heating, indicated that it can only originate from the portion of the HS in the amorphous phase participating in the H-bonding with the amorphous SS. The other observed peak at 1784 cm^−1^ probably originates from the non-associated acyl carbonyl of the HS in the amorphous phase (Figure 6).

The HS crystallinity can be calculated using only peak integral values of the peaks originating from the urethane carbonyl group at 1680 cm^−1^ (Figure 6a, cryst. HS, peak designated as *a*) and 1718 cm^−1^ (Figure 6a, amorph. HS, peak designated as *c*). However, since the acyl carbonyl peaks are a part of the HS and participate in the HS H-bonding, it seemed more accurate to combine the effect of the two carbonyl groups. Therefore, the HS crystallinity can be calculated as:

(3) $$HS_{cryst}=\frac{\int a+\int e}{\int a+\int b+\int e+\int f+\int g}\times 100\%$$

where *∫* stands for integral values of the peaks *a*, *b*, *f*, *g* and *h* as designated in Figure 6a.

The percentage of the SS crystallinity can be calculated as given in the Equation 4.

(4) $$SS_{cryst}=\frac{\int d}{\int d+\int e}\times 100\%$$

As expected, a higher percentage of HS crystallinity was observed for PAU1000 with respect to PAU1500 (Table 1).

**Table 1:** Hard and soft segment crystallinity of non-incubated PAUs determined by trans-FTIR.

Polymer	HS cryst^*^ (%)	SS cryst^**^ (%)
PAU1000-0	57.5	0.4
PAU1500-0	41.3	34.4

*Calculated according to Equation 3.

*Calculated according to Equation 3.

In general, PAUs were less microphase separated than the PUs based on the same molar mass of PCL but with the HS comprised of BDI and BDO (HS cryst.(PCL1000-PU) = 73% and HS cryst.(PCL1600-PU) = 61%) [26]. In addition to more hydrolysable HS, this lower degree of microphase separation renders PAUs more susceptible to hydrolysis.

### 2.4. Microphase Separation of PAUs Upon Degradation (ATR-FTIR)

Since PAU1000 was distinctly more microphase separated compared to the PAU1500, the morphology and the surface properties of PAU1000 were analyzed in more detail. ATR-FTIR, being a surface sensitive technique, provided information on the changes at the PAU1000 interface, which properties determine the protein deposition and the cell contact *in vivo* [14,15,27]. The change of surface properties upon degradation was followed by ATR-FTIR with utilization of the similar deconvolution method derived from the transmission measurements (Section 2.3). Due to optical complications, deconvolution of ATR-FTIR spectra can only be seen as a semi-quantitative technique. However, since we used this method to compare samples of the same polymer during degradation by identical manipulation of all the collected spectra, we consider the method to be acceptably credible. In order to emphasize the contribution of the acyl functionality to the hydrolysis of the PAUs, we also plotted the percentage HS crystallinity without the contribution of the acyl carbonyl peaks (denoted *e*, *f* and *g* in Figure 6a). This percentage was calculated according to the Equation 5.

(5) $$HS_{cryst}=\frac{\int a}{\int a+\int c}\times 100\%$$

where *∫* stands for integral values of the peaks *a* and *c* as designated in Figure 6a.

**Figure 7:**
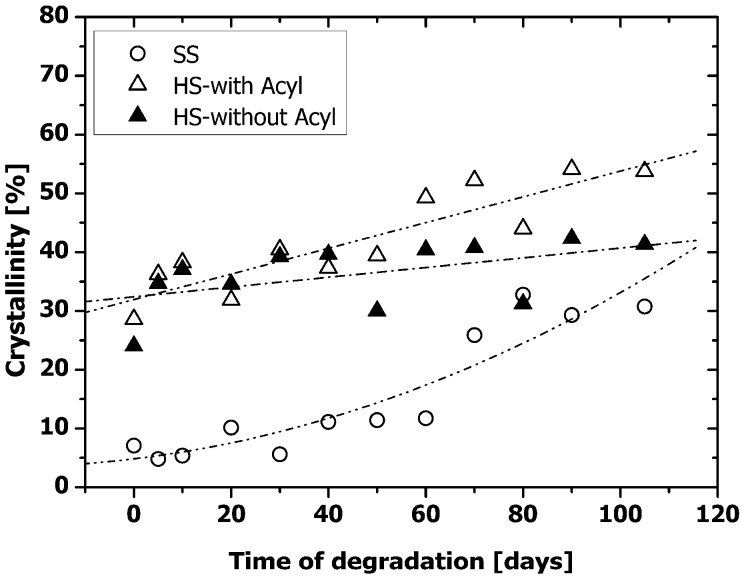
Percentage hard (cryst. HS) and soft segment crystallinity (cryst. SS) upon degradation of PAU1000. (○) Soft segment (SS) crystallinity (calculated according to Equation 4); (∆) hard segment crystallinity with the contribution of the acyl carbonyl peak (HS-with Acyl) (calculated according to Equation 3); (▲) hard segment crystallinity without contribution of the acyl carbonyl peak (HS-without Acyl) (calculated according to Equation 5).

**Figure 8:**
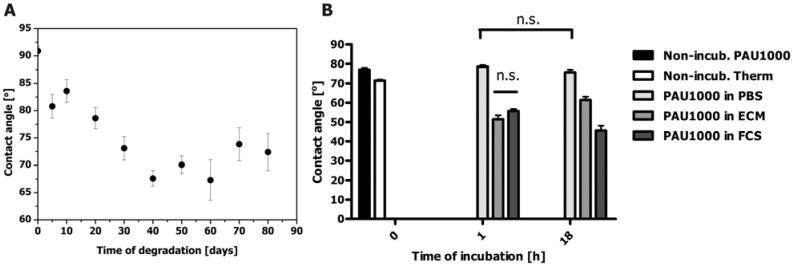
(**a**) Contact angle of PAU1000 during *in vitro* degradation; n = 5; data expressed as mean ± standard error of the mean; (**b**) Contact angle of PAU1000 exposed to biological fluids: Phosphate buffer saline (PAU1000 in PBS), endothelial cell culture medium (PAU1000 in ECM) and fetal calf serum (PAU1000 in FCS). References included: non-incubated PAU1000 (Non-incub. PAU1000) and non-incubated Thermanox^®^ (Non-incub. Therm). All the values, except where indicated (n.s.) were statistically significantly different (n = 5; data expressed as mean ± standard error of the mean).

Compared to the HS crystallinity of non-degraded PAU1000 in the bulk (Table 1), crystallinity of the HS on the surface was lower (Figure 8) (in the bulk: 57.5%, on the surface ~30%), which indicated that the polar, crystalline HS was initially located away from the surface. Although the overall crystalline SS content was low, it was predominantly present at the surface (in the bulk, Table 1: 0.4%, on the surface, Figure 7: ~5%), which has been found before in other similar segmented polyurethanes [5,28].

With degradation, the crystallinity of both SS and HS increased at the surface. As already mentioned, hydrolysis can only occur in the amorphous portions of the PAUs. If the products of degradation would not diffuse out, the crystallinity of both SS and HS would remain the same. However, we did observe mass loss (Figure 1), which can solely be ascribed to the hydrolysis of the amorphous SS and HS and the diffusion of the degradation products preferentially from the surface. The SS crystallinity at the surface increased for ~30% after 80 days of degradation (Figure 7), which was far more than observed in the bulk (Figure 5a). As already mentioned, PAUs degraded partially via surface erosion mechanism. Preferential hydrolytic chain scission at the surface probably induced higher chain mobility at the surface that allowed for SS crystallization. PCL crystallization upon degradation was also observed by *Lam et al.* [2] and *Antheunis et al.* [20]

Similar to the case of the SS crystallinity increase of PAU1000, the higher chain mobility at the surface of PAU1000 might also have allowed for better HS alignment, resulting in higher HS crystallinity at the surface relative to the bulk. Therefore, the increased crystallinity of both SS and HS upon degradation was most probably the result of both effects: Removal of the degradation products (mass loss) formed by ester and urethane bond hydrolysis in the amorphous portion of the PAUs, and the effect of the re-crystallization due to the higher mobility at the surface. The increased hydrogen-bonding capable hard HS content is expected to render the surface more hydrophilic, which is shown to further enhance cell adhesion [29,30].

### 2.5. Contact angle Measurements

The increase in overall crystallinity at the surface of PAU1000 upon *in vitro* degradation and surface erosion were expected to result in the increased surface roughness. An increase of surface roughness can be detected as the contact angle increase. However, hydrolysis created polar functional groups (carboxyl, hydroxyl or amide) and increased HS content at the surface of the degraded PAU1000, both contributing to an increased hydrophylicity, which was observed by a decrease of the contact angle (Figure 8a).

Somewhat lower contact angle values for non-incubated sample most probably originated from the different sample treatment in comparison to the rest of the data collected in the course of degradation (Figure 8a). After 50–60 days of degradation, the contact angle of PAU1000 was ~65°, which was close to optimal hydrophilicity conditions for cell adhesion and proliferation [29,30].

To model the biological environment of the potential PAU implant *in vivo*, the changes of the surface properties of PAU1000 in contact with biological fluids was investigated (Figure 8b). Coated on the Thermanox^®^ cover slip, the contact angle of the PAU1000 in a dry state was 78.0° ± 2.6° while the angle of the Thermanox^®^ cover slips was 71.1° ± 1.6° (Figure 8b). After incubation in PBS at 37 °C for 1 h, and 18 h the contact angle had slightly decreased, but after the incubation in ECM and FCS a noticeable increase of surface hydrophylicity, compared to the non-incubated sample, was observed, with the contact angle of PAU1000 incubated in FCS reaching 45.0° ± 7.2° (Figure 8b). This increase of PAU1000 hydrophylicity could explain the best HUVEC adhesion result at longer culture time. After 18 h incubation in ECM, the PAU1000 surface contact angle was 60°, which is, according to the literature, an optimal value for the best cell adhesion [29]. Most probably surface hydrophobicity changed due to an increase in hard segment content on the PAU1000 surface as a result of the interaction of hard segment with water and proteins at the interface in combination with products of the surface erosion process.

Upon implantation, PAU1000 performance is expected to be a result of a complex interplay between the material surface properties, protein deposition and a type of cells involved.

### 2.6. Cytotoxicity (MTS Assay)

In this study, we performed a reliable and robust method of determining cytotoxicity of the novel polyacylurethane PAU1000. Mitochondrial activity of PK-84 cells cultured in the medium containing extracts from PAU1000 was similar to both positive control and the blank, which was set to 100%, *i.e*., non-toxic, showing that PAU1000 was non-cytotoxic (Table 2).

**Table 2:** Cytotoxicity determination. Cytotoxicity of PAU1000 was comparable to clinical grade polyurethane Pellethane^®^. Controls were Latex (cytotoxic) and medium (non-toxic) (n = 4, data expressed as average ± SD).

[Test material	Cell survival [%]
PAU1000	86 ± 6
Pellethane^®^	91 ± 6
Latex	38 ± 1
Medium	104 ± 13

### 2.7. Haemocompatibility

**Figure 9:**
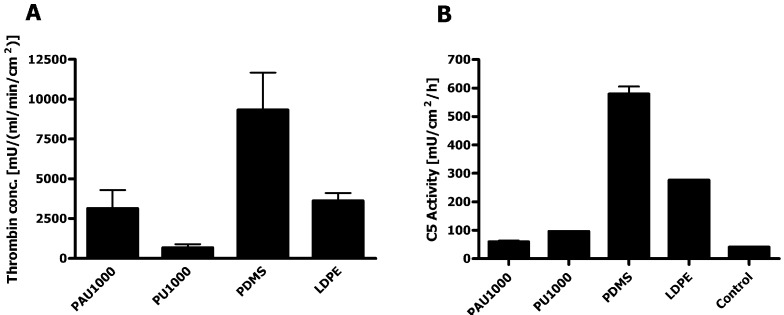
Haemocompatibility assessment. (**a**) Thrombin generation assay; (**b**) C5 complement convertase activity. Control materials used for comparison: 1,4-butanediisocyanate based polyurethane (PU1000), polydimethylsiloxane (PDMS) and low density polyethylene (LDPE) as the Gold standard (mean values of duplicate measurements ± SD).

The rate of thrombin generation by PAU1000 was similar to low density polyethylene, which is lowly thrombogenic, and much lower than polydimethylsiloxane, which served as *i.e.*, thrombogenic control material (Figure 9a). The other PCL-based PU, PU1000, was the least thrombogenic of all the materials tested. Since both the clotting cascade and platelet phospholipids were involved in this test, the results are clinically relevant. The good haemocompatibility renders PAU1000 a promising candidate for cardiovascular applications, e.g., bioartificial vascular grafts.

Complement convertases are formed by incubating biological fluids, blood or blood products with biomaterials, allowing the activation of the complement system and binding of complement convertases to the biomaterial surface. Surface activity of the C5 convertase indicates generation of C5a fragments, which are strong anaphylactic and chemotactic components and initiation of C5b-9, the terminal complement complex with cytotoxic capacity. Activation of the complement cascade in the presence of PAU1000 was measured through the activity of complement C5 convertase on the material surface. In this respect, PAU1000 only minimally activated complement compared to the other control biomedical materials (Figure 9b). Both PUs induced very low complement convertase activity. Therefore, the low C5 convertase activity recorded in the experiment with PAU1000 would be predicted to induce only a minimal inflammatory reaction *in vivo*.

### 2.8. HUVEC Adhesion to PAU1000

**Figure 10:**
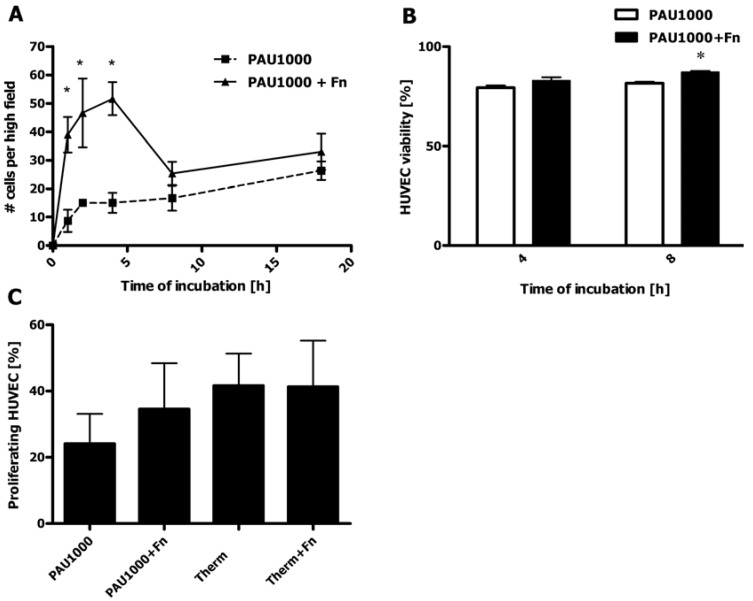
Adhesion and proliferation of endothelial cells (HUVEC) on PAU1000. (**A**) HUVEC adhesion to PAU1000 (■) and Fn-coated PAU1000 (▲) monitored up to 18 h. (n = 3; 6 images per cover slip, data expressed as mean ± standard error of the mean; asterisks indicate significant differences *p < 0.05); (**B**) Viability test of HUVEC during adhesion to PAU1000 and Fn-coated PAU1000 (PAU1000+Fn) at 4 and 8 h (n = 2; 6 images per cover slip, data expressed as mean ± standard error of mean; asterisks indicates significant difference *p < 0.05 ); (**C**) HUVEC proliferation after 25 h culture on PAU1000, Fn-coated PAU1000, Thermanox^®^ cover slips (Therm) and Fn-coated Thermanox^®^ cover slips (Therm+Fn) (n = 2; 5 images per cover slip, data expressed as mean ± standard error of the mean).

Adhesion of human umbilical vein endothelial cells (HUVEC) to PAU1000 was monitored for 18 h. Based on the hydrophobic nature of PAU1000, we expected only a low adhesion of HUVEC to the PU. However, the difference in contact angle values between PAU1000 (78.0° ± 2.6°) and Thermanox^®^ cell culture treated cover slips (71.1° ± 1.6°) was similar. Thus, in this setting hydrophobicity was not the only parameter governing the cell adhesion. We assume that the presence of carboxyl functionality on the surface of Thermanox^®^ cover slips rendered the surface more cell-adhesive. In order to further enhance cell adhesion, we included samples that were additionally coated with fibronectin. Fibronectin possesses integrin binding motifs such as the RGD (Arg-Gly-Asp) sequence that could augment the attachment of HUVEC [31,32]. Initially, the number of adhered cells increased regularly with time, irrespectively of Fn-coating, with Fn-coated PAU1000 cover slips providing faster cell adhesion for the period up to 4 h (Figure 10a).

HUVEC cultured on PAU1000 exhibited a steady increase in the number of adhered cells, resulting in a similar degree of adhered HUVEC on both Fn-coated and bare PAU1000. In order to elucidate the observed phenomenon of decrease of cell adhesion between the points 4 h and 8 h, the viability was measured using Trypan blue dye exclusion. The number of viable cells that adhered after incubation was at both time points more than 80% (Figure 10b), which was similar to the other time points (data not shown). Although the cells remained viable throughout the test period, some had detached from the Fn-coated slips after 8 h of culture. Since Fn is hydrophobic, a property that does not depend on the nature of the underlying substrate [33], the hydrophobic interaction of Fn with PAU1000 is strong. These strong hydrophobic interactions could have caused adverse conformational changes in the Fn molecules that impaired cell adhesion. Furthermore, the Fn-coating might have been partially lost during prolonged culturing, thus exposing the cells to the less adhesive bare surface of the PAU1000. In the later stages of incubation, adhering HUVEC started producing their own adhesive extracellular matrix molecules such as collagen and fibronectin (data not shown) [34]. This cellular activity might be the reason why with prolonged culture time (overnight), the difference between the HUVEC adhesion to bare PAU1000 and Fn-coated PAU1000 was diminished (Figure 10a).

A possible explanation for low cell adhesion to PUs could be that the PU surface exposed to plasma proteins is mostly covered with proteins that are generally considered non-adhesive. Fibrinogen is initially adhered, but it is competed for deposition on the polyurethane surface with albumin, high-density lipoprotein and high molecular weight kininogens (HMWK), which all do not favor cell binding [35]. The dynamics of the rearrangement and replacement of proteins on the PU surface is determined by the hydrophobicity and the degree of polyurethane micro-phase separation [10,34,36,37]. This influences protein mobility and rearrangement for better recognition by cell integrins [34]. Proteins interact with the polyurethanes via hydrophobic interactions (soft segment domains) and/or hydrogen bonding (hard segment). Highly hydrophobic substrates bind more protein as compared to more hydrophilic substrates. The hard segments are reported to bind fibrinogen via hydrogen bonds [36]. Being more adhesive, the hard segment is also known to interact with platelets and lead to their activation [28,37]. To contribute to the complexity of polyurethane-protein and cell interaction, a polyurethane surface is also dynamic and might rearrange to promote better adhesive properties by exposure of hard segments. A dry PU surface, being in contact with air, which is hydrophobic, has soft segments mostly exposed [5]. Upon immersion in water, more polar hard segments capable of hydrogen bonding migrate to the surface, making it more hydrophilic. The decrease in contact angle was observed for PAU1000 upon contact with biological fluids (Figure 8).

### 2.9. HUVEC Proliferation on PAUs

Even though the rate of HUVEC adhesion may appear low, longer incubation experiments of 25 h revealed that both bare PAU1000 and Fn-coated PAU1000 allowed for significant HUVEC proliferation, reaching almost 50% cellular confluence already after 25 h of culture, which was similar to the Thermanox^®^ cover slips (Figure 10c). That indicates that fast and efficient *in vitro* endothelialization of grafts fabricated from PAU1000 is feasible. The additional coating with Fn appeared to further increase the number of proliferating cells on PAU1000, although it had little influence on Thermanox^®^ cover slips.

## 3. Experimental Section

### 3.1. Materials

*ε*-Caprolactone (CL) was obtained from Union Carbide (Terneuzen, The Netherlands) and was purified by distillation under reduced pressure from calcium hydride (CaH_2_). Terephthaloyl diisocyanate was synthesized from terephthalamide, obtained from TCI (Japan) and oxalyl chloride from Sigma-Aldrich (Zwijndrecht, The Netherlands) using a modified method of Tsuge *et al.* [38]. Analytical grade 1,4-butanediol (BDO) was purchased from Sigma-Aldrich (Zwijndrecht, The Netherlands) and purified by distillation from 3 Å mole sieves under reduced pressure. Chloroform, diethyl ether, dimethyl formamide and n-hexane as analytical grade solvents were purchased from Acros Organics (Geel, Belgium) and were used without further purification. Sodium azide was purchased from Sigma-Aldrich (Zwijndrecht, The Netherlands) and used as received.

Polyacylurethane films were cast from chloroform onto PFA Petri dishes (perfluoroalkoxy polymer resin) obtained from Bergof (Florida, USA).

Thermanox^®^ cover slips which were purchased from NUNC^™^ (Roskilde, Denmark).

Glutaraldehyde was purchased from Sigma-Aldrich (Zwijndrecht, The Netherlands). RPMI medium and fetal calf serum (FCS) were obtained from Cambrex Bio Science (Verviers, Belgium). CellTiter 96^®^ Aqueous One Solution A was purchased from Promega Corporation (Madison, WI, USA). Polyurethane Pellethane^®^ was a gift from Dow Chemicals (Midland, MI, USA).

The following materials were used in the haemocompatibility study: Polydimethylsiloxane (PDMS) from Eriks (Alkmaar, The Netherlands), low-density polyethylene (LDPE) ET311350 from Goodfellow (Cambridge, UK), thrombin chromogenic substrate S_2238_ and chromogenic substrate S_2527_ from Chromogenix (Milano, Italy).

In cell adhesion and proliferation tests the following materials have been employed: Diamidino-2-phenylindol-dihydrochlorid (DAPI) from Sigma-Aldrich (Germany), Triton X-100 from Sigma (St. Louis, USA), polyclonal antibody rabbit-anti Ki-67 from Nova Castra Laboratory (Newcastle, UK), Tween 20 from Sigma-Aldrich (Zwijndrecht, The Netherlands), Avidin/Biotin blocking kit from Vector Laboratories Inc. (CA, USA), streptavidin-FITC from DAKO (The Netherlands) and Citifluor API from Agar Scientific (Essex, UK).

### 3.2. Synthesis of Polyacylurethanes

Polyacylurethanes (PAUs) were synthesized by a slightly modified method of Heijkants *et al.* [8]. The synthesis was performed in two steps: 1) oligodiol synthesis and 2) polymerization of the oligodiol with terephthaloyl diisocyanate (TPHDI) (Scheme 1). The oligodiols, poly(*ε*-caprolactone) (PCL) diols of three different lengths (1000, 1500 and 2000 g/mol) were prepared by thermal polymerization employing 1,4-butanediol as an initiator without the use of any catalyst at 150 °C for 7 days under argon [26]. The second step was performed in the Micro Twin Extruder. Briefly, a powder mixture of PCL (molar mass = 1000 g/mol) (approximately 7.0 g; 7.0 mmol) and TPHDI (0.75 g; 3.5 mmol) was fed to a micro-extruder at 65 °C. Subsequently, the extruder was heated up to 130 °C and another portion of TPHDI (0.75 g) was added. The average polymerization time was 6 minutes; the resulting polymer was collected, purified by precipitation in diethyl ether from chloroform solution, and dried in a vacuum stove at 40 °C. The reaction scheme is depicted in Scheme 1.

### 3.3. Preparation of Polymer Films

Polymer films were prepared by casting a 2 w/v% polyurethane solution in chloroform onto either ID 8 cm PFA Petri dishes or Thermanox^®^ cover slips (treated side up) at room temperature. Both the freestanding films and the coated slips were washed in a Soxhlet apparatus utilizing n-hexane as the extraction solvent for 16 h in order to remove any surface contaminants such as potentially present silicones. Finally, the materials were dried and stored in the vacuum stove at 40 °C until further use.

In some of the experiments, PAU-coated Thermanox^®^ cover slips were additionally coated with fibronectin (Fn). In brief, 250 μL of 1 weight% solution of recombinant human Fn in PBS was placed in wells (24-wells culture plate) containing PAU-coated slips and incubated at 37 °C for 30 min. The excess of Fn-solution was aspirated and the adhered fibronectin was cross-linked with 0.5% glutaraldehyde (GA) solution in PBS at room temperature for 15 min. In order to remove all the GA remains, all the coated samples were extensively washed with PBS and endothelial cell culture medium (ECM medium) formulated in our laboratory. The endothelial culture medium (ECM) consisted of RPMI 1640 supplemented with 20% heat-inactivated FSC, 2 mM L-glutamine, 5 U/mL heparin, 100 IE/mL penicillin and 100 μg/mL endothelial growth factor supplement extracted from bovine brain.

### 3.4. In Vitro Degradation Set-Up

Films obtained as described above were cut in quarters and weighed. Subsequently, each piece was placed in a glass container and 100 mL of phosphate buffer (solution in water, pH = 7.2; Sigma-Aldrich, Zwijndrecht, The Netherlands) solution, containing 0.02 wt% of sodium azide, was added. Labeled and well-closed containers were placed in a dark water bath at 37 °C. At predetermined time points, starting from 5 up to 80 days, samples (n = 2 per time point) were taken and rinsed with distilled water and blotted gently with tissue paper to remove surface water. Subsequently, the samples were dried in a vacuum oven at 40 °C. The weight was monitored until it reached a constant value.

### 3.5. Mass Loss

The mass loss of PAU-films upon *in vitro* degradation was determined by weighing the samples (one quarter of the film) at predestined time points and calculated as follows:

(6) $$Mass\;loss=\frac{\left(m_{o}-m_{d}\right)}{m_{o}}\times 100\%$$

Where *m_o_* is the dry mass before incubation and *m_d_* is the dry mass after degradation.

### 3.6. Molar Mass

The molar masses of the PAUs after synthesis and purification, as well as the molar masses of samples (n = 2 films per time point) upon degradation were determined by gel permeation chromatography (GPC) utilizing a Waters 600 Powerline system, equipped with 2 mixed-C Pl-gel 5 μ columns employing dimethyl formamide (DMF) with 0.01 M LiBr as eluent at 70 °C. The data analysis was done using conventional calibration with polystyrene standards accompanied by in-house software. Number average molar mass (*M_n_*) data (average of two samples per time point) were fitted by using the OriginPro 7.5 software.

### 3.7. Thermal Properties of PAUs

Thermal properties of PAUs as polymerized and at different time points of degradation were measured with a differential scanning calorimeter Q 1000 from TA Instruments. The samples with masses varying between 7–10 mg were heated from −85 °C to + 150 °C with a rate of 10 °C/min. The data collected during the first heating run were analyzed utilizing the TA Instruments software version 4.0.

### 3.8. Fourier-Transform Infra Red Spectroscopy (FTIR)

All the infrared spectral manipulation was performed using the Opus v.4.2 software package (Bruker Optik GmbH).

Infrared transmission measurements were done on films cast on KBr pellets from chloroform solution using a Bruker IFS88 spectrometer equipped with a MCT-A detector at the resolution of 2 cm^−1^. The KBr pellets were measured horizontally using the Bruker infrared microscope accessory. Five hundred scans were recorded per spectrum. Temperature was varied from room temperature to 120 °C.

The carbonyl absorption region from 1850 cm^−1^ to 1575 cm^−1^ was deconvoluted by fixing the peak position and allowing for peak intensity, width and shape to be optimized by the Opus software. The Levenberg-Marquardt algorithm was used in curve fit optimization. The calculated residual RSM fitting error was always <0.009.

ATR-FTIR was done using a Bruker IFS88 spectrometer equipped with a Golden Gate (Graseby Specac) single reflection ATR accessory. Spectral resolution was 4 cm^−1^ and 50 scans were taken per spectrum.

### 3.9. Contact angle Measurements

Surface properties of PAU1000 were assessed during the *in vitro* degradation up to a period of 80 days and upon short-term exposure to biological fluids. For the latter, PAU1000-coated Thermanox^®^ cover slips were placed in the wells of a 24-well culture plate and incubated in PBS, ECM supplemented with 20% FCS and 100% FCS at 37 °C for 1 h, and 18 h. In both cases, after incubation the samples were rinsed by means of spraying distilled water against both sides of the samples. The samples were blotted with tissue paper, free-standing film or cover slips were fixed to glass slides by double-sided adhesive tape and contact angle was measured using a sessile drop method using a Krüss Drop Shape Analysis System DSA 10.

### 3.10. MTS Cytotoxicity Assay

To perform cytotoxicity assays, a fibroblast cell line PK84 was cultured in RPMI medium containing 10% FCS. Cells seeded in a density of 5,000 cells/well were exposed to an extract of PAU1000, obtained by shaking the material overnight in culture medium at 37 °C. After 48 h 20 μL of CellTiter 96^®^ Aqueous One Solution A was added to each well and the absorbance intensity was recorded at 490 nm after 90 min of culture. Pellethane^®^ and latex were used as negative and positive control, respectively.

### 3.11. Haemocompatibility of PAU

Two different methods were employed to assess the blood compatibility of PAU1000: Thrombin generation assay and Complement convertase activity. Another biomedical polyurethane developed in our laboratory by Heijkants *et al.* [26] (1,4-butanediisocyanate-based (BDI) hard segment and PCL-based soft segment) with the same PCL length as PAU1000, and commonly used reference materials were included in the experiments.

The formation of thrombin in the presence of the biomaterial was determined by means of the Thrombin Generation Assay (TGA) as developed by Haemoscan (Groningen, The Netherlands). Polydimethylsiloxane (PDMS) and low-density polyethylene (LDPE) were used as positive and negative control, respectively. Thrombin generation was obtained in citrate plasma depleted of fibrinogen. Materials (surface area 0.5 cm^2^) were incubated in duplicate in 350 μL plasma in polyethylene tubes for 15 min at 37 °C. Then CaCl_2_ (30 mM) and phospholipids were added, gently mixed and after 1, 2, 4 and 6 min, 10 μL of the incubation mixture was diluted in 490 μL ice cold 25 mM Tris-HCl buffer to stop further thrombin formation or inhibition. These diluted samples were incubated at 37 °C with 3 mM thrombin chromogenic substrate S_2238_ for 20 min. The optical density of the yellow color was measured at 405 nm in a micro titer plate reader from Powerwave 200 Bio-Tech Instruments (Winooski, Vermont). A calibration curve was made with known concentrations of thrombin in Tris buffer.

Surface-bound C5 convertase was determined on 1 cm^2^ material after incubation in porcine plasma for 15 min at room temperature (CCA, Haemoscan, Groningen, The Netherlands). After incubation, the samples were rinsed and incubated in chromogenic substrate S_2527_) diluted in TRIS buffer at room temperature in the dark for 24 h. Thereafter, the optical density was determined at 405 nm in a microplate reader.

### 3.12. HUVEC Adhesion and Viability on PAU1000

Human umbilical vein endothelial cells (HUVEC) were isolated and cultured as previously described [39]. In short, the cells were cultured on endothelial culture medium (ECM; see Section 3.3) and 1% gelatin coating at 37 °C and 5% CO_2_. Polyurethane (PAU1000)-coated Thermanox^®^ cover slips and additionally fibronectin (Fn)-coated cover slips were placed in 24-well plates and HUVEC (130.000/cm^2^) were seeded in each well. After 18 h of culture, adhered cells were fixed in 2% paraformaldehyde (PFA) in PBS at room temperature for 20 min. Cells were stained with DAPI in PBS for 30 min. The cell number was determined by fluorescence microscopy utilizing Leica DC 300F apparatus (Wetzlar, Germany). For the viability test, HUVEC (150.000/cm^2^) in ECM were seeded onto either PAU1000-coated cover slips, or Fn-coated PAU1000 cover slips, and cultured for 4 and 8 h. The adhered cells were detached with trypsin and the viability was assessed using Trypan blue staining.

### 3.13. HUVEC Proliferation on PAU1000

The HUVEC proliferation was evaluated upon culture on PAU1000, Fn-coated PAU1000, bare Thermanox^®^ cover slips and Fn-coated Thermanox^®^ cover slips for 25 h. After culture, the non-adhered cells were removed by washing and the attached cells were fixed with 2% PFA in PBS and stored dry at 4 °C. For the staining, samples were thawed, dried and additionally fixed with 2% PFA in PBS. Fixed cells were permeabilized with 0.5% Triton X-100 in PBS. Samples were then incubated with polyclonal antibody rabbit-anti Ki-67 (1:500) at room temperature for 90 min. Subsequently, cells were washed with 0.05% Tween 20 in PBS. Endogenous avidin and biotin were blocked with Avidin/Biotin blocking kit for 15 min each. Samples were then incubated with the secondary antibody goat anti rabbit biotin in DAPI/PBS solution at room temperature in the dark for 30 min. Thereafter, cover slips were incubated with streptavidin-FITC in DAPI/PBS at room temperature in the dark for 30 min. All incubation steps were followed by appropriate washing steps. The cover slips were transferred and fixed onto glass slides, and mounted in Citifluor API. The cover slips were examined by immunofluorescence microscopy using a Leica DMRXA microscope and Leica Software of Leica Microsystems (Wetzlar, Germany). At least six images were recorded per cover slip and the attached cells were counted.

Due to the high auto fluorescence of uncoated PAU1000, we also detached the cells after overnight culture and cytospotted the cells on glass slides for 5 min and speed of 500 rpm using Shendon, Cytospin 3 apparatus. The staining procedure for Ki67 was the same as previously mentioned.

### 3.14. Statistical Analysis

A statistical analysis was performed by two-way ANOVA followed by Bonferroni post hoc analysis using GraphPad Prism software v4 (San Diego, California, USA). Values of p < 0.05 were considered statistically significant.

## 4. Conclusions

Polyacylurethanes (PAUs) degraded *in vitro* via combined bulk and surface erosion mechanisms. Due to a faster hydrolysable hard segment based on terephthaloyl diisocyanate and lower degree of microphase separation, PAUs degraded much faster *in vitro* than comparable PUs with the same polyester (PCL) soft segment. Predominant chain scission at the surface led to different surface properties of PAUs with respect to the bulk. Surface erosion and increased chain mobility at the surface resulted in the increase of both soft and hard segment crystallinity upon degradation. Generation of the polar groups upon hydrolysis and the increase of the hard segment content on the surface probably led to the increase in hydrophilicity, which further renders PAU1000 potentially cell adhesive. PAU1000 (molar mass of the oligocaprolactone soft segment = 1000 g/mol) can be recommended as a potential scaffold material to be used in regenerative medicine due to its optimal *in vitro* behavior. PAU1000 behaved as a non-toxic and blood-compatible biomaterial. In addition, PAU1000 supported adhesion and proliferation of human umbilical vein endothelial cells. Taken together, these results support the application of PAU1000 in the field of soft tissue repair as a temporary degradable scaffold.
